# Combining the strength of genomics, nanoparticle technology, and direct intraductal delivery for breast cancer treatment

**DOI:** 10.1186/bcr3656

**Published:** 2014-05-17

**Authors:** Wei Wen Teo, Saraswati Sukumar

**Affiliations:** 1 Breast Cancer Program, Sidney Kimmel Comprehensive Cancer Center, Johns Hopkins University School of Medicine, 1650, Orleans Street, CRB1/143, Baltimore, MD 21231, USA

## Abstract

A large number of genes are altered in cancer cells. Often, reversal or inhibition of just one of these alterations leads to death of the cancer cells. Technological advances in multiple areas are necessary to potentiate clinical translation of these findings. In a recent article, Brock and colleagues reported that overexpressed HOXA1 is a critical event in tumor progression in a mouse mammary tumor model. They developed HOXA1-small interfering RNA nanoparticles and achieved effective therapeutic doses by delivering them intraductally through the nipple to the site of the tumor and at the same time circumvented the systemic immune response. This study strengthens the concept of targeting overexpressed genes by using small interfering RNA and bypassing systemic immunity through local intraductal delivery.

## Introduction

Whole genomic analysis has identified a large number of genes that are overexpressed in cancer cells; reversal of mechanisms leading to overexpression often leads to death of the cancer cells. Selective gene inhibition by the small interfering RNA (siRNA) strategy has fired the imagination of drug developers. This excitement is based on the ability of siRNAs to enter the cell and interfere with virtually every targetable transcript that codes for intracellular proteins, and even non-coding RNA. However, keeping the siRNA intact during systemic transport, preventing an immune response against it, and getting it to its target organ at levels needed for successful therapy have presented large barriers.

## The article

In the article by Brock and colleagues [1], the authors bring two concepts together to overcome obstacles in siRNA therapy. First, using tissue-specific microarray analysis in a transgenic mammary tumor model, they constructed a gene regulatory network to identify key promoters active during tumor formation. Among other candidates, the transcription factor HOXA1 was found to be significantly overexpressed during cancer progression. Second, using lipidoid nanoparticles as a carrier, they delivered siRNA targeting HOXA1 to mouse mammary glands via intraductal (i.duc) injection, thereby ensuring successful targeting and bypassing systemic immune response. The siRNA-conjugated nanoparticles, when injected through the nipples of C3 (1)-SV40Tag transgenic mice, significantly reduced the incidence of spontaneous mammary tumors.

They also observed that siRNA-mediated depletion of HOXA1 in two other mouse mammary tumor cell lines resulted in more acinar (hollow) lumen formation of cells grown in matrigel. Findings in this cell culture model, commonly used to mimic preneoplastic ductal carcinoma *in situ* (DCIS) in humans, suggested that HOXA1 may be a key mediator in the early stages of breast tumor formation. Oncomine database analysis strengthened their hypothesis since HOXA1 was found to be overexpressed in human breast cancers. To silence HOXA1 in mammary tissues *in vivo*, they injected virgin C3 (1)-SV40Tag mice with nanoparticle-siRNA complex bi-weekly through the nipple for 9 weeks, starting when the mice were 12 weeks of age. Whereas all the control mice developed tumors, 75% of HOXA1 siRNA-nanoparticle-treated mice remained tumor-free at 21 weeks. Most noteworthy is that i.duc treatment with siRNA in the transgenic mice did not cause local tissue damage or any obvious systemic side effect. When performed multiple times, i.duc injection can cause leakage of material into normal tissue. Therefore, the lack of deleterious consequences is an important positive feature of the nanoparticles.

## Viewpoint

Advances in next-generation sequencing and bioinformatics are continuing to inform us of the steps from cancer initiation to progression. Among transcripts that are differentially expressed in breast cancer, those that are highly expressed in cancer cells are theoretically targetable by siRNA. The hopes pinned on siRNAs stem from the fact that they are more specific than other cancer therapies; indeed, they can even discriminate between species that have single-nucleotide differences [2,3]. As a result, phenotypes caused by gain-of-function mutations and gene translocations are also potentially reversible with siRNA-based therapy.

I.duc siRNA therapy would benefit women with atypical ductal hyperplasia or DCIS and women at high risk of developing breast cancer because of a family history of mutated BRCA1/2 or other familial breast cancer genes. Could the preneoplastic lesions present in the entire ductal tree be accessed for treatment through the i.duc route? As early as 2002, Sivaraman and colleagues [4] demonstrated the feasibility of accessing rat ducts through the nipple for treatment. We used i.duc injection of slow-release liposomal doxorubicin (Doxil) to successfully eliminate preneoplasias in both the HER/2 neu transgenic mouse and carcinogen-induced rat mammary tumor models [5] and to delay carcinoma formation by using curcumin [6]. We [7] and others [8] also demonstrated the feasibility and safety of the i.duc approach by using Doxil in women undergoing mastectomy for breast cancer. However, the use of DNA-synthesis-targeted drugs carries the risk of iatrogenic cancer in the distant future. Although there are clear species differences between humans and mice, laboratory-bred female mice developed cancer when doxorubicin [9] or fluorouracil (our unpublished findings) was injected intraductally, with a latency of nearly a year. On the other hand, agents such as siRNA and similar biologic therapies may not carry these risks.

The article by Brock and colleagues [1] has firmly established the feasibility of using HOXA1 siRNA-nanoparticles as an effective cancer preventive/therapeutic agent and their delivery into the ducts through the nipple as a means of treating mammary preneoplasias in the preclinical setting. However, as pointed out by the authors, this study does not offer evidence that HOXA1 is a candidate gene that is overexpressed in DCIS [10] or that HOXA1 overexpression in DCIS predicts progression to invasive cancer [11]. Several laboratories, including ours, are engaged in comparative genomic analysis of DCIS that does, or does not, progress to invasive cancer; these studies will likely shed light on the importance of HOXA1 in the progression of DCIS to invasive cancer in the near future. Secondly, the number of i.duc injections that were performed to observe a reduction in tumor incidence presents a barrier to clinical translation. Although the intactness of the ducts is retained for up to four injections, our experience shows that the likelihood of collapsing ducts is high in mice if injections continued multiple times. Pharmacokinetic studies in the future could offer indications of longer-lasting effects of the siRNA-nanoparticles and thus the need for less frequent injections. This will ensure feasibility of clinical trials testing this concept. Despite these considerations for future translation of the work to the clinic, the work has undoubtedly demonstrated the power of discovery of overexpressed genes that are critical for development of the mammary tumor, the development of the siRNA strategy, and successful implementation of the approach by using intraductal administration to circumvent the immune system and to directly deliver effective doses of the agent at the desired site of the tumor, while sparing the other organs.

## Abbreviations

DCIS: Ductal carcinoma *in situ*; Doxil: Liposomal doxorubicin; i.duc: Intraductal; siRNA: Small interfering RNA.

## Competing interests

The authors declare that they have no competing interests.
